# *Chryseobacterium schmidteae* sp. nov. a novel bacterial species isolated from planarian *Schmidtea mediterranea*

**DOI:** 10.1038/s41598-021-90562-3

**Published:** 2021-05-26

**Authors:** Luis Johnson Kangale, Didier Raoult, Eric Ghigo, Pierre-Edouard Fournier

**Affiliations:** 1 IRD, AP-HM, SSA, VITROME, Aix-Marseille Univ, Marseille, France; 2 Institut Hospitalo-Universitaire Méditerranée Infection, IHU-Méditerranée-Infection, Aix-Marseille Université, 19-21 Boulevard Jean Moulin, 13385 Marseille cedex 05, France; 3 IRD, AP-HM, MEPHI, Aix-Marseille Univ, Marseille, France; 4 Special Infectious Agents Unit, King Fahd Medical Research Center, King Abdulaziz University, Jeddah, Saudi Arabia; 5 TechnoJouvence, 19-21 Boulevard Jean Moulin, 13385 Marseille cedex 05, France

**Keywords:** Microbiology, Bacteriology

## Abstract

Marseille-P9602^T^ is a *Chryseobacterium*-like strain that we isolated from planarian *Schmidtea mediterranea* and characterized by taxono-genomic approach. We found that Marseille-P9602^T^ strain exhibits a 16S rRNA gene sequence similarity of 98.76% with *Chryseobacterium scophthalmum* LMG 13028^T^ strain, the closest phylogenetic neighbor. Marseille-P9602^T^ strain was observed to be a yellowish-pigmented, Gram-negative, rod-shaped bacterium, growing in aerobic conditions and belonging to the *Flavobacteriaceae* family. The major fatty acids detected are 13-methyl-tetradecanoic acid (57%), 15-methylhexadecenoic acid (18%) and 12-methyl-tetradecanoic acid (8%). Marseille-P9602 strain size was found from genome assembly to be of 4,271,905 bp, with a 35.5% G + C content. The highest values obtained for Ortho-ANI and dDDH were 91.67% and 44.60%, respectively. Thus, hereby we unravel that Marseille-P9602 strain is sufficiently different from other closed related species and can be classified as a novel bacterial species, for which we propose the name of *Chryseobacterium schmidteae* sp. nov. Type strain is Marseille-P9602^T^ (= CSUR P9602^T^ = CECT 30295^T^).

## Introduction

Using genotypic, chemotaxonomic and phenotypic characteristics of members of *Flavobacterium* and *weeksella* genus allowed revising the classification of the novel C*hryseobacterium* genus^1^ with *Chryseobacterium gleum* type strain^2^. Several genus members were isolated from soil, plant, waste water, fish, sewage, sludge, lactic acid beverage, oil, contaminated soil, and clinical samples^3–12^. Some species of this genus such as *Chryseobacterium indologenes*, *Chryseobacterium oranimense* and *Chryseobacterium gleum* are responsible for human pathologies^13,14^; others are involved in the production of natural bioactive substances such as prebiotics, antioxidants, and proteases^15–17^. *Chryseobacterium* cells wereobserved to be gram-negative, non-motile, non-spore-forming rods, with parallel sides and rounded ends. Typically, these cells are 0.5 mm wide and 1 to 3 mm long^1^. All strains grow at 30 °C; most strains grow at 37 °C. Growth on solid media is typically pigmented (yellow to orange). Colonies were observed to be translucent (occasionally opaque), circular, convex, or low convex, smooth, and shiny, with entire edges^1^. In this study, we used the genomic and taxonomy strategy that combines phenotypic assays and genome sequencing^18–21^ to further characterize a *Chryseobacterium*-like bacterial strain isolated from planarian *Schmidtea mediterranea* species. *S. mediterranea* platyhelminth is a zoophage invertebrate living in freshwater like ponds, lakes, and rivers^22^. This flatworm is a model organism for regeneration, because of its unique capacity to regenerate after amputation^23^, as well as to investigate host–pathogen interaction^24–26^.

## Materials and methods

### Culture of Schmidtea mediterranea

*S. mediterranea* animals are asexual (clonal line ClW4), kept in laboratory for 10 years and fed with calf liver, maintained in filtered tap water at 19 °C as previously described^27^.

### Isolation and identification of bacteria from Schmidtea mediterranea

Before experiments, animals were starved for two weeks, washed in sterile water and then one worm was inoculated in Buffered Charcoal Yeast Extract (BCYE) (Oxoid Deutschland GmbH, Wesel, Germany), Luria Bertani (LB) and 5% sheep blood-enriched Columbia agar (bioMérieux, Marcy l’étoile, France) and incubated at 19, 28 and 37 °C. Bacterial colonies were identified by MALDI-TOF-MS (Microflex spectrometer; Bruker Daltonics, Bremen, Germany)^28^, as previously described^27^. Briefly, a colony was likely identified at the species level for a score ≥ 2.0; probably identified for a score between 1.99 and 1.7, but not identified for a score < 1.7.

### Sequencing, assembly, and annotation

First, using EZ1 automate and DNA tissue kit (Qiagen, Hilden, Germany), bacterial genomic DNA was extracted and then quantified using a Qubit assay (Life Technologies, Carlsbad, CA, USA) at 0.2 ng/µl. Second, bacterial genomic DNA was prepared and sequenced using Mate-Pair strategy with a Miseq sequencer (Illumina, San Diego, CA, USA)^29^. Next, sequencing reads were assembled using Spades software (Galaxy version 3.12.0 + galaxy1)^30^ and genomic annotation was obtained using Prokka (Rapid Prokaryotic Genome Annotation)^31^. Finally, taxonomic assignation was done by BLASTn search performed against nr database. A sequence similarity threshold of 98.65% by comparison with the phylogenetically closest species with standing in nomenclature was used to delineate a putative novel species^32^.

### Phylogenetic analysis, and genomic comparison

Phylogenetic relationships were inferred from comparison of 16S rRNA gene sequences using MEGAX (version10.1) software^33,34^. Sequences were aligned using MUSCLE algorithm setup with default parameters, and numbers at the nodes were percentages of bootstrap values obtained by repeating the analysis 1000 times to generate a majority consensus tree. Only bootstrap values ≥ 50% were retained. For the Phylogenetic tree based on the core genes, we generated a core-gene alignment using Roary 3.13.0^35^ with 70% identity. We obtained an alignment of 1535 core genes from which we inferred a phylogenetic tree using FastTree 2.1.10^36^. Degrees of genomic similarity were evaluated using the GGDC^37^ (http://ggdc.dsmz.de/ggdc.php#) and Orthologous Average Nucleotide Identity^38^ (https://www.ezbiocloud.net/tools/orthoani, OrthoANI Tool version 0.93.1) softwares. Comparison COG functional categories were carried out using Blast P (E-value 10-3, coverage 0.7 and identity percent 30%) against clusters of orthologous groups (COG) database.

### Phenotypic characteristics

Growth of Marseille-P9602 strain and *Chryseobacterium scophthalmum* LMG 13028^T^ strain (purchased to DSMZ) (ATCC 700,039 = CCM 4109 = CCUG 33,454 = CIP 104,199 = DSM 16,779 = MM1)^1,39^ was attempted at various temperatures such as 4, 19, 28, 30, 37 and 45 °C in 5% sheep blood-enriched Columbia agar (bioMérieux) under anaerobic atmosphere using GasPak EZ generators (Becton–Dickinson, Maryland, USA), as well under aerobic atmosphere. Strain ability to sporulate was investigated by thermal shock. Briefly, bacteria were exposed at80 °C temperature for 30 min and then bacterial growth was assessed for 4 days. The capacity to growth under various salinity (0, 20, 40, 50, 60, 80 and 100 g of NaCl/l) and pH conditions (5, 5.5, 6, 6.5, 7.5, 8.5, 9 and 10) was also investigated. Gram staining and motility of fresh colonies were observed using a DSM1000 photonic microscopy (Leica Microsystems, Nanterre, France) with an ocular of 10 × and 40 × objective lens. Bacterial structure was defined using a scanning electron microscopy (Hitachi SUV5000) (Hitachi High-Technologies Corporation, Tokyo, Japan). Enzymatic activities such as catalase and oxydase activities were analysed with a BBL DrySlide following manufacturer's instructions (Becton Dickinson, Le Pont de Claix, France). API strips (API ZYM^40–42^, API 20NE^43,44^, API 20E^45,46^ and API 50CH^47–50^, bioMérieux) were used to study strains biochemical characteristics.

### Antibiotic susceptibility of Marseille-P9602 strain

Bacterial susceptibility to benzylpenicillin, amoxicillin, ampicillin, ceftriaxone, imipenem, ciprofloxacin, amikacin, gentamicin, streptomycin, daptomycin, doxycycline, metronidazole, rifampicin, fosfomycin, vancomycin and tigecycline was assessed using *E*-tests and a 0.5 McFarland concentration of Marseille-P9602 and LMG 13028^T^ strains. MICs were read at the point of intersection between the developed elliptical zone of inhibition and the test strip. Interpretation of the MICs was carried out according to NCCLS recommendations for bacterial isolates grown aerobically^51^.

### Analysis of cellular fatty acids of strain Marseille-P9602

Cellular fatty acid methyl ester (FAME) analysis was performed by GC/MS for both Marseille-P9602 and LMG 13028^T^ strain. Fatty acid methyl esters were prepared as described by Sasser^52^ and GC/MS analysis was realized as previously described^53^. Briefly, Marseille-P9602 and LMG 13028^T^ strains were inoculated in 5% sheep blood-enriched Columbia agar and incubated at 28 °C. Fatty acid methyl esters were separated using an Elite 5-MS column and monitored by mass spectrometry (Clarus 500—SQ 8 S, Perkin Elmer, Courtaboeuf, France). Spectral database search was performed using MS Search 2.0 operated with the Standard Reference Database 1A (NIST, Gaithersburg, USA) and the FAMEs mass spectral database (Wiley, Chichester, UK).

## Results and discussion

### Phylogenetic analysis and genomic comparison

**Figure 1:**
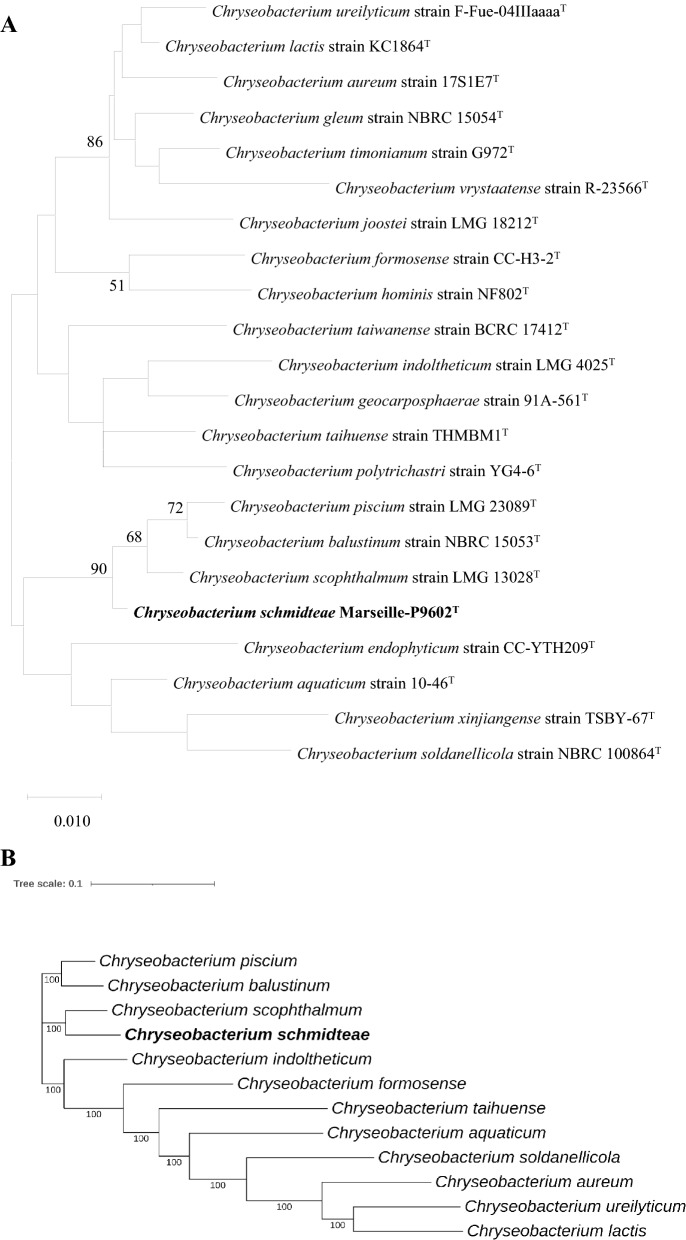
Phylogenetic tree and Core-genome. (**A**) Phylogenetic tree based on 16S rRNA sequence comparison highlighting the position of Marseille-P9602 strain relative to other closely related species. Only bootstrap values ≥ 50% were shown. (**B**) Core-genome-based phylogenetic relationships of Marseille-P9602 strain relative to other closely related species.

**Figure 2:**
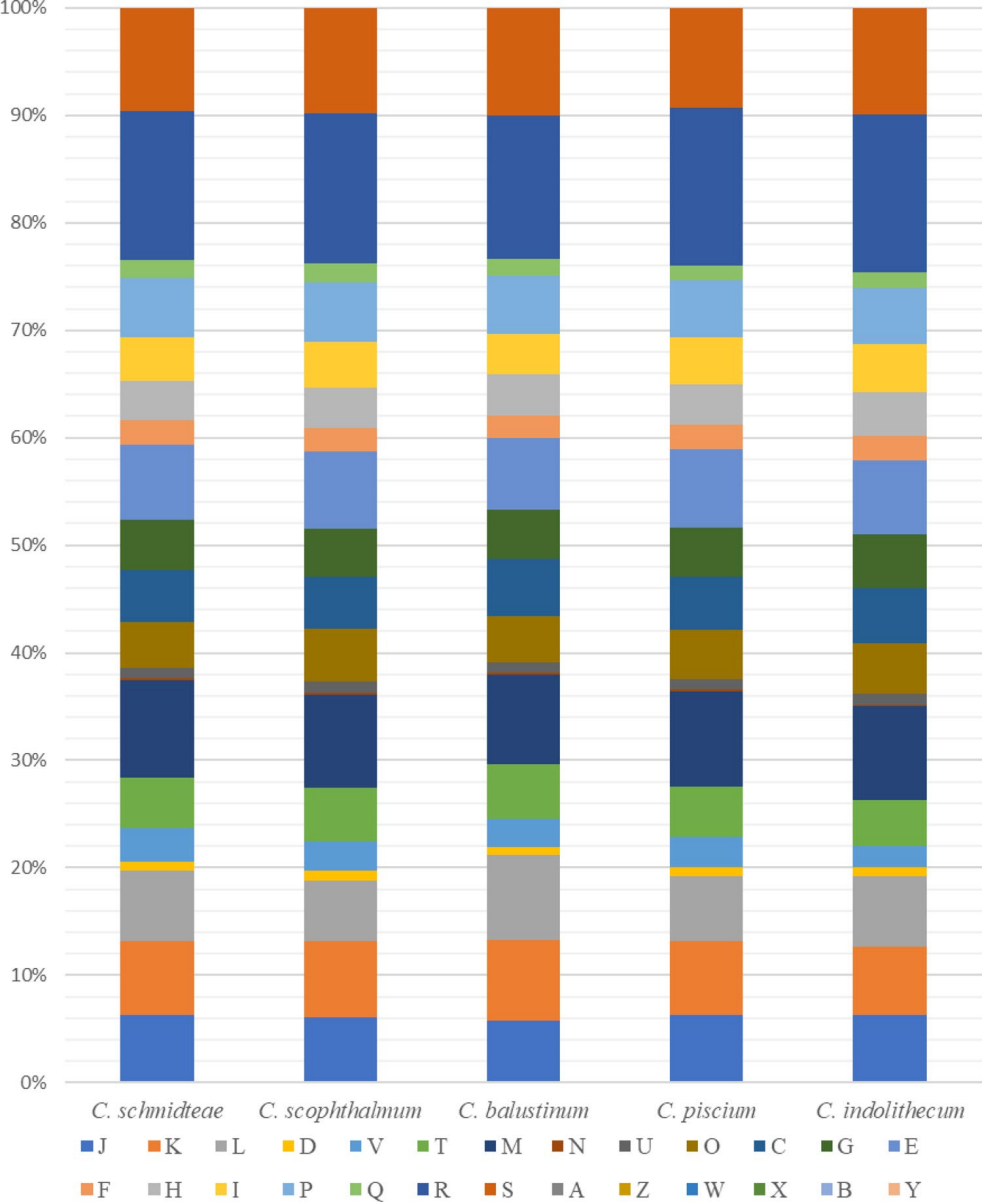
Functional annotation of predicted gene according to the COGs comparison of COGs of Marseille-P9602 species with phylogenetically related species of the genus *Chryseobacterium.* [A] RNA processing and modification; [B] Chromatin structure and dynamics; [C] Energy production and conversion; [D] Cell cycle control, cell division, chromosome partitioning; [E] Amino acid transport and metabolism;[F] Nucleotide transport and metabolism; [G] Carbohydrate transport and metabolism; [H] Coenzyme transport and metabolism; [I] Lipid transport and metabolism; [J] Translation, ribosomal structure, and biogenesis; [K] Transcription; [L] Replication, recombination, and repair; [M] Cell wall/membrane/envelope biogenesis; [N] Cell motility; [O] Posttranslational modification, protein turnover, chaperones; [P] Inorganic ion transport and metabolism; [Q] Secondary metabolites biosynthesis, transport, and catabolism; [R] General function prediction only; [S] Function unknown; [T] Signal transduction mechanisms; [U] Intracellular trafficking, secretion, and vesicular transport; [V] Defense mechanisms; [W] Extracellular structures; [X] Mobilome: prophages, transposons; [Y] nuclear structure [Z] Cytoskeleton.

The gene 16S rRNA sequence from Marseille-P9602 strain was observed to be 1513 bp-long. A sequence similarity calculation using BLASTn search in the nr database indicated that the closest relatives of Marseille-P9602 strain are *Chryseobacterium scophthalmum* LMG 13028^T^ strain^1,39^, *Chryseobacterium piscium* LMG 23089^T^ strain^54^, *C. balustinum* NBRC 15053^T^ strain^1^, *C. indoltheticum* LMG 4025^T^ strain^55^, *C. taihuense* THMBM1^T^ strain^55^, *C. ureilyticum* F-Fue-04IIIaaaa^T^ strain^7^, *C. aquaticum* 10-46^T^ strain^6^, *C. lactis* KC1864^T^ strain^8^, *C. soldanellicola* NBRC 100864^T^ strain^9^, *C. formosense* CC-H3-2^T^ strain^56^*,C. aureum* 17S1E7^T^ strain^57^, *C. hominis* NF802^T^ strain^58^, *C. timonianum* G972^T^ strain^59^ *C. polytrichastri* YG4-6^T^ strain^10^, *C. echinoideorum* CC-CZW010^T^ strain^60^, *C. xinjiangense* TSBY-67^T^ strain^61^, *C. endophyticum* CC-YTH209^T^ strain^62^, *C. taiwanense* BCRC 17412^T^ strain^63^, *C. vrystaatense* R-23566^T^ strain^64^, *C. joostei* LMG 18212^T^ strain^65^, *C. geocarposphaerae* 91A-561^T^^66^, and *C. gleum* NBRC 15054^T^ strain^2^, whose similarity values, coverage and accesssion strain numbers are shown in Table 1. Therefore, Marseille-P9602 strain belongs to *Chryseobacterium* genus^1^ within the *Flavobacteriaceae* family^67^ and the *Bacteroidetes* phylum^68^ (Table 2). The 16S rRNA-based phylogenetic tree showed that Marseille-P9602, *C. scophthalmum* LMG 13028^T^, *C. piscium* LMG 23089^T^ and *C. balustinum* NBRC 15053^T^ strains form a monophyletic group (Fig. 1A). Core genome tree showed that strains are different from each other (Fig. 1B). The genomic sequence from Marseille-P9602 strain was assembled into 56 contigs for a total size of 4,276,845 bp (Cover, 56x; N_50_, 151,068; L_50_, 9) with a 33.5% G + C content. A total of 3881 predicted protein-coding genes were identified, along with 9 rRNAs, 67 tRNAs, 1 tmRNA and 1 repeat region; and this genome was compared with other closely related *Chryseobacterium* genomes (Table 3). Based on the Digital DNA-DNA hybridization values (dDDH) obtained using GGDC software, Marseille-P9602 strain values ranged from 21.40% with *C. aureum* and *C. lactis* to 44.60% with *C. scophthalmum* (Table 3). These values were below the 70% threshold recognized for the delimitation of bacterial species. Ortho-ANI values of Marseille-P9602 strain ranged from 76.65% *C. aureum* to 91.67% with *C.* scophthalmum, which is lower than the 95% threshold used to distinguish species (Table 3). These values of genomic comparison showed that Marseille-P9602 strain is probably a novel species in the *Chryseobacterium* genus. The distribution of genes in COG functional categories is presented in Fig. 2 and Table 4. Few differences were observed between these species. In addition, by comparison of the genomes of Marseille-P9602 strain and of the 11 closest species, we highlighted 100 specific and unique genes to the Marseille-P9602 strain (Supplementary data S1). Taken together, these results confirm that Marseille-P9602 strain belongs to a separate *Chryseobacterium* species.

**Table 1:** The taxonomic assignment obtained by a BLASTn search in the nr database.

	Marseille-P9602 (LR797929)		
Name	Cover (%)	Identity (%)	N° Accession
*Chryseobacterium scophthalmum* strain LMG 13028^T^	97	98.76	NR_025386.1
*Chryseobacterium piscium* strain LMG 23089^T^	98	98.36	NR_042410.1
*Chryseobacterium balustinum* strain NBRC 15053^T^	97	98.75	NR_113721.1
*Chryseobacterium indoltheticum* strain LMG 4025^T^	96	97.98	NR_042926.1
*Chryseobacterium taihuense* strain THMBM1^T^	99	96.62	NR_109542.1
*Chryseobacterium ureilyticum* strain F-Fue-04IIIaaaa^T^	99	96.53	NR_042503.1
*Chryseobacterium aquaticum* strain 10-46^T^	96	97.34	NR_042642.1
*Chryseobacterium lactis* strain KC1864^T^	97	97.01	NR_126256.1
*Chryseobacterium xinjiangense* strain TSBY-67^T^	100	96.09	NR_131771.1
*Chryseobacterium soldanellicola* strain NBRC 100864^T^	97	96.89	NR_113952.1
*Chryseobacterium formosense* strain CC-H3-2^T^	98	96.57	NR_036872.1
*Chryseobacterium aureum* strain 17S1E7^T^	99	96.02	NR_170500.1
*Chryseobacterium hominis* strain NF802^T^	99	96.08	NR_042517.2
*Chryseobacterium timonianum* strain G972^T^	99	96.02	NR_164881.1
*Chryseobacterium polytrichastri* strain YG4-6^T^	99	96.07	NR_134710.1
*Chryseobacterium echinoideorum* strain CC-CZW010^T^	99	95.95	NR_145657.1
*Chryseobacterium endophyticum* strain CC-YTH209^T^	98	96.30	NR_156142.1
*Chryseobacterium taiwanense* strain BCRC 17412^T^	99	95.61	NR_043715.1
*Chryseobacterium vrystaatense* strain R-23566^T^	98	96.17	NR_042370.1
*Chryseobacterium joostei* strain LMG 18212^T^	98	96.17	NR_025387.1
*Chryseobacterium geocarposphaerae* strain 91A-561^T^	97	96.54	NR_133727.1
*Chryseobacterium gleum* strain NBRC 15054^T^	97	96.40	NR_113722.1

Marseille-P9602 strain has a high sequence similarity, but a sequence cover lower^38^, with *Chryseobacterium scophthalmum* strain LMG 13028^T^, *Chryseobacterium piscium* strain LMG 23089^T^, *Chryseobacterium balustinum* strain NBRC 15053^T^ and *Chryseobacterium indoltheticum* strain LMG 4025^T^.

**Table 2:** Classification and general features of Marseille-P9602 strain.

Property	Term
Current classification	Domain: Bacteria ^69^
	Phylum: Bacteroidetes ^68,70,71^
	Class: Flavobacteriia ^72^
	Order: Flavobacteriales ^73^
	Family: *Flavobacteriaceae* ^67^
	Genus name: *Chryseobacterium* ^1^
	Species name: *Schmidteae*
	Specific epithet: *Chryseobacterium schmidteae*
	Type strain: Marseille-P9602
Species status	sp. nov
Gram stain	negative
Cell shape	rod-shaped
Motility	Non-motile
Sporulation	non-spore-forming
Temperature range for growth	4–30
Temperature optimum	20
pH range for growth	6.5–9
pH optimum	7.5
pH category	Neutro-alkalophilic
Lowest NaCl concentration for growth	0
Highest NaCl concentration for growth	12 g/L
Salinity optimum	5 g/L
O2 conditions for strain testing	Yes
Catalase	Negative
Oxydase	Positive
Habitat	Planarians
Biotic relationship	Planarian Microbiota

**Table 3:** Main genomic characteristics of Marseille-P9602 and other closely related *Chryseobacterium* species.

				Marseille-P9602		
Name	Size (bp)	Contig	CDS	DDH	ANI	Refseq
**Marseille-P9602**	4.276.845	56	3881	100	100	NZ_CAESCJ010000000
*C. scophthalmum*	4.468.393	9	4085	44.60	91.67	NZ_FSRQ00000000.1
*C. piscium*	4.319.169	158	3953	38.60	90.56	NZ_QNVS00000000.1
*C. balustinum*	4.545.564	24	4152	39.10	89.62	NZ_UAVR00000000.1
*C. indoltheticum*	4.253.895	1	3901	33.40	87.42	NZ_CP033929.1
*C. taihuense*	3.685.675	1	3358	22.70	79.27	NZ_LR215974.1
*C. ureilyticum*	5.183.225	27	4743	21.50	77.04	NZ_FTOL00000000.1
*C. aquaticum*	3.813.178	21	3443	23.90	80.89	NZ_LLYZ00000000.1
*C. lactis*	5.618.212	1	5030	21.40	76.89	NZ_CP033924.1
*C. soldanellicola*	4.136.421	9	3734	22.80	78.87	NZ_FNKL00000000.1
*C. formosense*	4.432.606	14	4006	26.00	82.58	NZ_FPAP00000000.1
*C. aureum*	5.069.854	1	4472	21.40	76.65	NZ_CP034661.1

OrthoANI values calculated using OAT software^38^. dDDH values obtained by comparison of all studied genomes using GGDC, formula 2 (DDH Estimates Based on Identities/HSP length).

**Table 4:** Functional annotation of predicted genes according to the COGs.

Code	Value	Description
**Information storage and processing**		
[J]	154	Translation, ribosomal structure and biogenesis
[A]	0	RNA processing and modification
[K]	170	Transcription
[L]	163	Replication, recombination and repair
[B]	0	Chromatin structure and dynamics
**Cellular processes and signaling**		
[D]	20	Cell cycle control, cell division, chromosome partitioning
[Y]	0	Nuclear structure
[V]	77	Defense mechanisms
[T]	116	Signal transduction mechanisms
[M]	222	Cell wall/membrane/envelope biogenesis
[N]	6	Cell motility
[Z]	0	Cytoskeleton
[W]	0	Extracellular structures
[U]	23	Intracellular trafficking, secretion, and vesicular transport
[O]	105	Posttranslational modification, protein turnover, chaperones
[X]	0	Mobilome: prophages, transposons
**Metabolism**		
[C]	119	Energy production and conversion
[G]	114	Carbohydrate transport and metabolism
[E]	173	Amino acid transport and metabolism
[F]	55	Nucleotide transport and metabolism
[H]	91	Coenzyme transport and metabolism
[I]	101	Lipid transport and metabolism
[P]	136	Inorganic ion transport and metabolism
[Q]	39	Secondary metabolites biosynthesis, transport and catabolism
**Poorly characterized**		
[R]	342	General function prediction only
[S]	237	Function unknown

Functional annotation of Marseille-P9602 predicted genes according to the COGs database.

### Phenotypic analysis and biochemical characteristics

**Figure 3:**
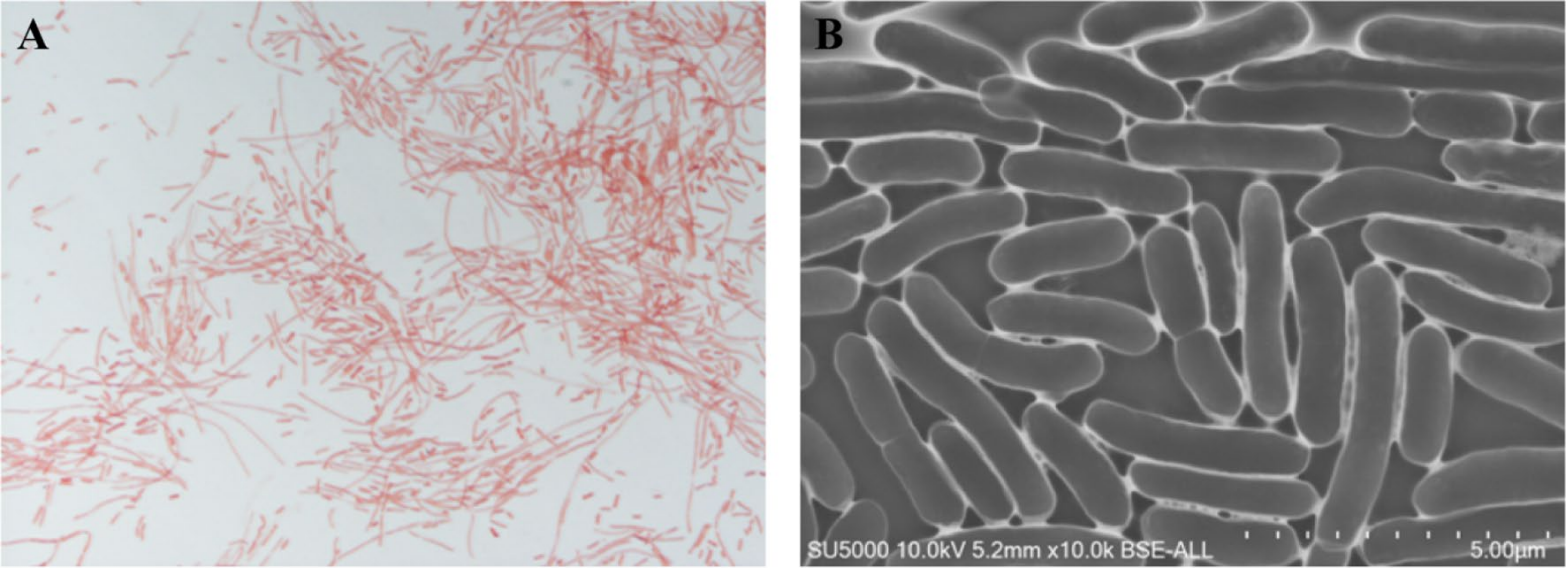
Micrograph of Marseille-P9602 strain. (A) Micrograph of Marseille-P9602 strain after Gram staining, (B) Transmission electron microscopy micrograph of Marseille-P9602 strain.

Marseille-P9602 strain was isolated on COS agar after 2 days at 28 °C in aerobic atmosphere at pH 7.5. We observed that Marseille-P9602 strain grows at temperatures ranging from 4 to 30 °C in aerobic atmosphere and at pH values ranging from 6.5 to 9 (Neutro-alkalophilic bacterium). In contrast, LMG 13028^T^ strain grows at pH 6. Marseille-P9602 strain grows at salinity concentrations lower than 12 g of NaCl/l; however, in contrast, LMG 13028^T^ strain needs a NaCl concentration lower than 25 g/l. After 4 days culture on COS agar, Marseille-P9602 strain colonies were observed to be yellowish, small (0.4 mm median diameter), circular with a convex shape and smooth. Bacterial cells (Fig. 3) are Gram-negative (Fig. 3A), rod-shaped, non-spore-forming bacilli and non-motile, but without any flagellum. Their mean length and width are 3.15 µm and 0.66 µm, respectively (Fig. 3B). Marseille-P9602 strain was found to be oxidase positive and catalase negative. Bacterial metabolism was characterized using API 50CHB/E, API 20NE, API Zym and API 20E strips (Table 5). Marseille-P9602 strain differs from *C. scophthalmum*, *C. indoltheticum*, *C. piscium*, and *C. balustinum* regarding catalase, α-glucosidase, inositol and urea.

**Table 5:** Biochemical characteristics of Marseille-P9602 and phylogenomically related species.

Properties	1	2	3	4	5
Gram-staining	−	−	−	−	−
Sporulation	−	−	−	−	−
Growth temperature range (°C)	4–30	4–30	5–30	5–35	5–37
Aerobic growth	+	+	+	+	+
Source	Planarian	S. maximus	sea	fish	fish
Colony colour	Yellowish	Yellowish	Yellow	Yellow	Yellow
Catalase	−	+	+	+	+
Oxydase	+	+	+	+	+
**Enzyme activity (API ZYM)**:					
Alkaline phosphatase	+	+	+	+	NA
Esterase (C4)	+	+	−	NA	−
Esterase lipase (C8)	−	−	+	NA	NA
Lipase (C14)	+	+	−	NA	−
Leucine arylamidase	+	+	+	NA	NA
Valine arylamidase	−	−	+	NA	NA
Cystine arylamidase	+	+	+	NA	−
Trypsin	−	−	−	NA	−
α-chymotrypsin	+	+	+	NA	−
Acid phosphatase	+	+	+	+	NA
Naphtol-AS-BI-phosphohydrolase	−	−	+	NA	NA
α-galactosidase	−	−	−	NA	NA
β-galactosidase	−	−	−	NA	−
β-glucuronidase	−	+	−	NA	NA
α-glucosidase	−	+	+	NA	NA
β-glucosidase	+	+	−	NA	+
N-acetyl-β-glucosaminidase	−	−	+	NA	NA
α-mannosidase	−	−	−	NA	NA
α-fucosidase	+	+	−	NA	NA
**Assimilation of (API 50 CH /B)**					
Glycérol	−	−	NA	NA	NA
Erythritol	−	−	NA	NA	−
d-arabinose	−	−	−	NA	−
l-arabinose	−	−	−	NA	−
d-ribose	−	−	NA	NA	−
d-xylose	−	−	−	−	NA
l-xylose	−	−	−	−	NA
d-adonitol	−	−	−	−	NA
Methyl-βd-xylopyranoside	−	−	NA	−	NA
d-galactose	−	−	−	−	NA
d-glucose	+	+	+	−	+
d-fructose	+	+	+	NA	NA
d-mannose	+	+	+	+	NA
l-sorbose	+	−	NA	NA	NA
l-rhamnose	−	+	−	−	NA
Dulcitol	−	−	−	−	NA
Inositol	+	−	−	−	NA
d-mannitol	−	+	−	−	+
d-sorbitol	−	−	NA	NA	−
Methyl-αd-mannopyranoside	−	−	NA	NA	NA
Methyl-αd-glucopyranoside	−	−	NA	NA	NA
N-acetylglucosamine	−	−	−	NA	NA
Amygdalin	+	+	NA	NA	NA
Arbutin	−	−	NA	NA	NA
Esculin ferric citrate	+	+	+	+	NA
Salicin	−	−	−	NA	NA
d-cellobiose	−	−	−	−	−
d-maltose	−	−	+	−	−
d-lactose	−	−	−	−	+
d-melibiose	−	−	NA	−	NA
d-saccharose	−	−	NA	−	NA
d-trehalose	+	+	−	−	NA
Inulin	−	−	−	−	NA
d-melezitose	−	−	−	−	NA
d-raffinose	−	−	−	−	NA
Starch	−	−	−	−	-
Glycogen	−	−	−	NA	NA
Xylitol	−	−	NA	NA	NA
Gentiobiose	+	+	−	+	NA
d-turanose	−	−	NA	−	NA
d-lyxose	−	−	NA	−	NA
d-tagatose	−	−	NA	−	NA
d-fucose	−	−	NA	−	NA
l-fucose	−	−	NA	−	-
d-arabitol	−	−	NA	−	-
l-arabitol	−	−	NA	−	
Potassium gluconate	−	−	−	−	-
Potassium 2-ketoGluconate	−	−	NA	−	NA
Potassium 5-ketogluconate	−	−	NA	−	NA
**API 20E**					
l-lysin	**−**	**−**	NA	NA	NA
l-ormithin	**−**	**−**	NA	NA	+
Trinatriumcitrat	**−**	**−**	NA	NA	NA
Natriumthiosulfat	**−**	**−**	+	NA	**-**
l-tryptophan	+	+	NA	NA	NA
Indole production	+	+	+	NA	+
Natriumpyruvat	+	**−**	NA	NA	NA
**API 20NE**					
Potassium nitrate	+	+	−	+	+
l-arginine	−	−	+	NA	+
Urea	−	+	+	+	-
Gelatin	+	+	+	+	NA
N-acetyl-glucosamine	−	−	NA	−	NA
Capric acid	−	−	−	−	-
Adipic acid	−	−	−	−	NA
Malic acid	−	−	−	−	NA
Trisodium citrate	−	−	−	−	-
Phenylacetic acid	−	−	−	−	-

Taxa: 1, Marseille-P9602; 2, *C. scophthalmum*; 3, *C. indoltheticum*; 4, *C. piscium*; 5, *C. balustinum.* The results presented for 1 and 2 are those obtained in the present study. The results presented for 3, 4 and 5 were completed using previously published studies^54,55,74^. positive (+); negative (−); NA, non-available. Marseille-P9602 strain differs from *C. scophthalmum*, *C. indoltheticum*, *C. piscium*, and *C. balustinum* regarding catalase, α-glucosidase, inositol and urea.

### Antibiotic susceptibility

Marseille-P9602 strain growth is inhibited by benzylpenicillin, amikacin, amoxicillin, ampicillin, gentamicin, ciprofloxacin ceftriaxone, streptomycin, doxycycline, tigecycline, rifampicin, and vancomycin; but not by daptomycin, fosfomycin, and metronidazole (Table 6). We noticed that amikacin inhibits the growth of Marseille-P9602, but not LMG 13028^ T^ strain.

**Table 6:** Antimicrobial susceptibility and MIC values of Marseille-P9602 and *Chryseobacterium scophthalmum* LMG 13028^T^ strains.

Drug (Antibiotics)	CC µg/ml	Marseille-P9602 MIC	LMG 13028^ T^ MIC
Benzylpenicellin	0.016–256	12	8
Amikacin	0.016–256	8	> 256
Amoxicillin	0.016–256	128	48
Ampicillin	0.016–256	24	8
Gentamicin	0.64–1024	6	64
Ciprofloxacin	0.002–32	0.25	0.38
Ceftriaxone	0.016–256	12	12
Streptomycin	0.064–1024	2	32
Daptomycin	0.016–256	> 256	> 256
Doxycyclin	0.016–256	1	2
Tigecycline	0.016–256	3	4
Fosfomycin	0.064–1024	> 256	> 256
Metronidazole	0.016–256	> 256	> 256
Rifampicin	0.002–32	0.004	0.38
Vancomycin	0.016–256	48	24

*CC* Tested range of drug concentration in µg/ml (microgram/milliliter). *MIC* Minimum inhibition of concentration in µg/ml (microgram/milliliter).

### Cellular fatty acids analysis

The fatty acids 13-methyl-tetradecanoic acid (56.7%), 15-Methylhexadecenoic acid (18.1%), 12-methyl-tetradecanoic acid (7.5%), 3-methyl-butanoic acid (4.9%),3-hydroxy-15-methyl-Hexadecanoic acid (3.3%), Hexadecanoic acid (1.2%) and 11-methyl-Dodecanoic acid (1.2%) were detected in Marseille-P9602 strain. Trace (< 1%) of unsaturated and saturated fatty acids such as 15-methyl-Hexadecanoic acid, 9,12-Octadecadienoic acid, 12-methyl-Tridecanoic acid, Pentadecanoic acid, 9-Octadecenoic acid, 3-hydroxy-Hexadecanoic acid, Tetradecanoic acid, 9-Hexadecenoic acid, and Octadecanoic acid were detected. The fatty acid 3-hydroxy-13-methyl-Tetradecanoic was not detected in Marseille-P9602 strain, in contrast to *C. scophthalmum*, *C. indoltheticum*, *C. piscium*, and *C. balustinum* strains (Table 7).

**Table 7:** Cellular fatty acid composition of Marseille-P9602 strain compared with related species.

Fatty acids	Name	1	2	3	4	5
14:0	Tetradecanoic acid	tr	tr	–	–	–
15:0	Pentadecanoic acid	tr	–	–	–	–
16:0	Hexadecanoic acid	1.2	1.0	1.1	1.2	2
18:0	Octadecanoic acid	tr	–	–	–	–
16:0 3-OH	3-hydroxy-Hexadecanoic acid	tr	–	1.3	1.5	1
17:0 2-OH	2-hydroxy-Hexadecanoic acid	–	–	–	1.8	–
5:0 iso	3-methyl-butanoic acid	4.9	9.6	–	–	–
13:0 iso	11-methyl-Dodecanoic acid	1.2	tr	0.9	tr	tr
14:0 iso	12-methyl-Tridecanoic acid	tr	tr	–	–	–
15:0 iso	13-methyl-tetradecanoic acid	56.7	50.6	38.3	32.3	33
17:0 iso	15-methyl-Hexadecanoic acid	tr	1.1	1.2	tr	1
15:0 3-OH iso	3-hydroxy-13-methyl-Tetradecanoic acid	–	3.8	2.4	5.1	3
16:0 3-OH iso	3-hydroxy-13-methyl-hexadecanoic acid	–	–	tr	2.0	tr
17:0 3-OH iso	3-hydroxy-15-methyl-Hexadecanoic acid	3.3	3.8	16.2	19.0	17
15:0 anteiso	12-methyl-tetradecanoic acid	7.5	tr	2.7	5.3	tr
16:1ω7	9-Hexadecenoic acid	tr	tr	10.8	22.1	9
17:1ω9 iso	15-Methylhexadecenoic acid	18.1	20.9	18.7	4.7	27
18:2ω6	9,12-Octadecadienoic acid	tr	tr	–	–	–
18:1ω9	9-Octadecenoic acid	tr	tr	–	–	–
Unknown	Unknown fatty acid	4.5	6.6	1.2	–	2

Taxa: 1, Marseille-P9602; 2, *C. scophthalmum*; 3, *C. piscium*; 4, *C. indoltheticum*; 5, *C. balustinum*. The results presented for 1 and 2 were obtained in the present study. The results presented for 3, 4 and 5 were completed using previously published studies^54,55^. Analysis of the fatty acid methyl esters was performed by Gas liquid chromatography according to the instructions for the Microbial Identification System (MIDI). tr, Trace (< 1%); not detected (−); present (+); NA, data not available.

## Conclusion

Based on the results obtained by the taxono-genomic approach, we confirm that Marseille-P9602 strain belongs to a novel species from *Chryseobacterium* genus. We propose the name of *Chryseobacterium schmidteae* Marseille-P9602^T^ strain. To date, this novel strain has never been identified in any other environment.

### Species description

*Chryseobacterium schmidteae* (schmid.te'ae. N.L. gen. n. schmidteae of the planarian genus *Schmidtea*, from which Marseille-P9602 strain was isolated) is a bacterium belonging to the *Flavobacteriaceae* family within the Bacteroidetes phylum. Marseille-P9602^T^ type-strain was isolated on 5% sheep blood-enriched Columbia agar after 2 days at 28 °C in aerobic atmosphere at pH 7.5 from the microbiota of planarian *Schmidtea mediterranea*. Colonies were observed to be small, circular, smooth, yellowish and convex. Cells were found to be Gram-negative, rod-shaped, non-motile and non-spore-forming bacilli with negative catalase and positive oxydase activities. The major fatty acids were found to be 13-methyl-tetradecanoic acid, 15-Methylhexadecenoic acid, 12-methyl-tetradecanoic acid, 3-methyl-butanoic acid, 3-hydroxy-15-methyl-Hexadecanoic acid, 3-hydroxy-13-methyl-Tetradecanoic acid, Hexadecanoic acid and 11-methyl-Dodecanoic acid. It was observed to be positive for alkaline phosphatase, esterase (C4), lipase (C14), leucine arylamidase, cystine arylamidase, α-chymotrypsin, acid phosphatase, naphthol-AS-BI-phosphohydrolase, β-glucosidase and α-fucosidase, but negative for valine arylamidase, esterase lipase (C8), α-galactosidase, β-galactosidase, α-glucosidase, N-acetyl-β-glucosaminidase, α-mannosidase, and β-glucuronidase activities. It assimilates glucose, mannose, d-fructose, l-sorbose, amygdalin, inositol, esculin ferric citrate, gentiobiose and d-trehalose, but not glycerol, maltose, erythritol, d-arabinose, l-arabinose, d-ribose, d-xylose, l-xylose, d-adonitol, methyl-β d-xylopyranoside, d-galactose, l-rhamnose, Dulcitol, d-mannitol, d-sorbitol, methyl-αd-mannopyranoside, methyl-αd-glucopyranoside, N-acetylglucosamine, arbutin, salicin, d-cellobiose, d-lactose, d-melibiose, d-saccharose, inulin, d-melezitose, d-raffinose, glycogen, xylitol, d-turanose, d-lyxose, d-tagatose, d-fucose, l-fucose, d-arabitol, l-arabitol, Starch, potassium gluconate, potassium 2-ketogluconate, potassium and 5-ketogluconate. Positive reactions were observed for l-tryptophan, natrium pyruvat, indole production, potassium nitrate, and gelatin, but no reaction was detected for l-lysin, l-ormithin, trinatrium citrate, natrium thiosulfate, l-arginine, urea, N-acetyl-glucosamine, capric acid, malic acid, trisodium citrate, adipic acid, and phenylacetic acid. The genome of Marseille-P9602^T^ strain was found to be 4.271.905 bp-long with a 35.5% G + C content. The 16S rRNA gene and genome sequences were deposited in GenBank under the accession numbers LR797929 and CAESCJ000000000.1, respectively. Marseille-P9602^T^ type-strain was deposited in the CSUR strain collections under the numbers CSUR P9602 and CECT 30295.

### Nucleotide sequence accession number

16S rRNA gene sequence and genome sequence were deposited in GenBank under the accession numbers LR797929 and CAESCJ000000000.1, respectively. The raw data for the assembly were deposited in EMBL-EBI under the run accession ERR4143501 and the experiment accession ERX4110774.

### Deposit in culture collections

Marseille-P9602^T^ strain was deposited in the Collection de Souches de l'Unité des Rickettsies (CSUR) and Colección Española De Cultivos Tipo (CECT) strain collections under the numbers CSUR P9602 and CECT 30295, respectively.

## Supplementary information

Supplementary Information.

Supplementary Legends.
