# Publisher Correction: *Chryseobacterium schmidteae* sp. nov. a novel bacterial species isolated from planarian *Schmidtea mediterranea*

**DOI:** 10.1038/s41598-021-97051-7

**Published:** 2021-08-31

**Authors:** Luis Johnson Kangale, Didier Raoult, Eric Ghigo, Pierre-Edouard Fournier

**Affiliations:** 1 IRD, AP‑HM, SSA, VITROME, Aix-Marseille Univ, Marseille, France; 2 Institut Hospitalo‑Universitaire Méditerranée Infection, IHU‑Méditerranée‑Infection, Aix-Marseille Université, 19‑21 Boulevard Jean Moulin, 13385 Marseille cedex 05, France; 3 IRD, AP‑HM, MEPHI, Aix-Marseille Univ, Marseille, France; 4 Special Infectious Agents Unit, King Fahd Medical Research Center, King Abdulaziz University, Jeddah, Saudi Arabia; 5 TechnoJouvence, 19‑21 Boulevard Jean Moulin, 13385 Marseille cedex 05, France

Correction to: *Scientific Reports* 10.1038/s41598-021-90562-3, published online 26 May 2021

The original version of this Article contained a repeated error, where the reference number of the CECT strain “30295” was incorrectly given as “30,295”.

The original Article has been corrected.

