# Supplementary material for: Chryseobacterium schmidteae sp. nov. a novel bacterial species isolated from planarian Schmidtea mediterranea
Source: Sci Rep. 2021 May 26;11:11002. doi: 10.1038/s41598-021-90562-3 (PMC8155073; doi:10.1038/s41598-021-90562-3)
Supplement: Supplementary file 2 — Supplementary Legends. [file 41598_2021_90562_MOESM2_ESM.docx]

**Legends suplementary data S1:** list of genes present in all the bacterial strain used in this study, and list of specific genes present in the Marseille-P9602 strain
